# Prognostic value of kallikrein-related peptidase 12 (KLK12) mRNA expression in triple-negative breast cancer patients

**DOI:** 10.1186/s10020-020-0145-7

**Published:** 2020-02-07

**Authors:** Weiwei Gong, Yueyang Liu, Sarah Preis, Xiaocong Geng, Agnes Petit-Courty, Marion Kiechle, Alexander Muckenhuber, Tobias Dreyer, Julia Dorn, Yves Courty, Viktor Magdolen

**Affiliations:** 1 Clinical Research Unit, Department of Obstetrics and Gynecology, Technical University of Munich, Ismaninger Str. 22, 81576 Munich, Germany; 2 Department of Gynecology, Guangdong Provincial People’s Hospital and Guangdong Academy of Medical Sciences, Guangzhou, People’s Republic of China; 3 INSERM, U1100 - Centre d’Etude des Pathologies Respiratoires, Tours, France; 4 Institute of Pathology, Technical University of Munich, Munich, Germany

**Keywords:** KLK12, qPCR, mRNA, TNBC

## Abstract

**Background:**

The serine protease KLK12 belongs to the human fifteen-member family of kallikrein-related peptidases. Differential expression accompanied by either increased or decreased enzymatic activity has been linked to several diseases including cancer. Triple-negative breast cancer (TNBC) represents a very aggressive subgroup of breast cancer with high tumor recurrence rates and poor patient prognosis. Here, we quantified the KLK12 mRNA expression levels in tumor tissue of TNBC patients and analyzed their prognostic value.

**Methods:**

In the present study, KLK12 mRNA expression in tumor tissue of TNBC patients (*n* = 116) was determined by quantitative real-time PCR assay. The association of KLK12 mRNA levels with clinical parameters, and patients’ outcome was analyzed using Chi-square tests, Cox regression models and Kaplan-Meier survival analysis.

**Results:**

Positive, but low KLK12 mRNA levels were detected in about half of the cases (54 out of 116; 47%), the other samples were negative for KLK12 mRNA expression. No significant association was observed between KLK12 mRNA levels and clinicopathological variables (age, lymph node status, tumor size, and histological grade). In univariate Cox analyses, positive KLK12 mRNA expression was significantly associated with shortened disease-free survival (DFS; hazard ratio [HR] = 2.12, 95% CI = 1.19–3.78, *p* = 0.010) as well as overall survival (OS; HR = 1.91, 95% CI = 1.04–3.50, *p* = 0.037). In multivariable Cox analysis, including all clinical parameters plus KLK12 mRNA, the latter - together with age - remained an independent unfavorable predictive marker for DFS (HR = 2.33, 95% CI = 1.28–4.24, *p* = 0.006) and showed a trend towards significance in case of OS (HR = 1.80, 95% CI = 0.96–3.38, *p* = 0.066).

**Conclusions:**

Positive KLK12 expression is remarkably associated with shortened DFS and OS, suggesting that KLK12 plays a tumor-supporting role in TNBC.

## Background

Tissue kallikrein (KLK1) and the kallikrein-related peptidases (KLK2–15) genes encode for a subgroup of 15 homologous secreted serine proteases with trypsin- or chymotrypsin-like activities. They are co-localized within the chromosomal region 19q13.3–4 representing the largest contiguous family of protease genes in the human genome (Diamandis et al. 2000; Clements et al. 2001). In normal physiology, the KLK genes are expressed in various tissues and the encoded enzymes are notably known to be involved in the regulation of blood pressure and kidney function (KLK1), skin desquamation (KLK5, 7, 14), tooth enamel formation (KLK4), seminal liquefaction (KLK2, 3, 5, 14), and synaptic neural plasticity and brain function (KLK6, 8). Alterations in expression and activity of KLKs have been linked to several diseases, including skin and brain disorders, respiratory diseases, pathological inflammation, and cancer (Chao et al. 2010; Eissa and Diamandis 2008; Kalinska et al. 2016; Lenga Ma Bonda et al. 2018; Pampalakis and Sotiropoulou 2007; Shiosaka and Ishikawa 2011). The involvement of KLKs in multiple malignancies regarding tumor cell growth and regulation of epithelial-to-mesenchymal transition, angiogenesis, as well as tumor invasion and metastasis highlight the importance of these proteases in the promotion of cancer progression. However, a number of opposing effects have been documented for KLKs in different types of malignancies indicating that their actions depend on the tumor type and/or the tumor microenvironment. These divergent effects are often reflected in the unfavorable or favorable prognostic values found for the mRNA and/or protein expression levels (Avgeris et al. 2012; Borgono and Diamandis 2004; Filippou et al. 2016; Kryza et al. 2015). Therefore, assessment of the KLK expression profiles in a specific background may help to understand the molecular aspects of cancer progression in this context and to identify potential stratification biomarkers. Availability of such biomarkers will become a key trait for clinical practice in the future to facilitate drug development and treatment decision.

The present study focuses on the analysis of KLK12 mRNA expression in tumor tissue of triple-negative breast cancer (TNBC) patients. The KLK12 gene belongs to the kallikrein gene cluster (Yousef et al. 2000) and is expressed in a variety of normal tissue including colon, salivary glands, stomach, trachea and vagina (Shaw and Diamandis 2007). Deregulation of KLK12 expression has been observed in gastric (Li et al. 2017) and skin (Giusti et al. 2005, 2006) disorders, and in gastric (Li and He 2016; Zhao et al. 2012), salivary gland (Morrison et al. 2018), prostate (Memari et al. 2007), breast (Papachristopoulou et al. 2018; Talieri et al. 2012) and lung tumors (Planque et al. 2008; Swarts et al. 2013; Guillon-Munos et al. 2011). The currently known functions of the KLK12 protease are mainly related to angiogenesis. KLK12 induced cell growth of microvascular endothelial cells (MVEC) in skin (Giusti et al. 2005) as well as MVEC migration and capillary morphogenesis in skin and lung (Giusti et al. 2005; Guillon-Munos et al. 2011; Kryza et al. 2013, 2014, 2018). Beside its implications in angiogenesis, KLK12 may also play a role in proliferation and migration of cancer cells, as described for gastric cancer cells in vitro (Li and He 2016; Zhao et al. 2012). As the tumor microenvironment may modulate the biological consequences of KLK actions, it may be desirable to study the KLK12 expression in a homogeneous cohort comprising only one cancer subtype.

Therefore, for the present study, we established a cohort of 116 patients afflicted with triple-negative breast cancer (TNBC), which accounts for approximately 10–15% of all breast cancers and is defined by the absence of estrogen receptor (ER), progesterone receptor (PR), and HER2 overexpression. TNBC is a very aggressive subgroup of breast cancer: it is more often diagnosed at younger age, in higher stage, with higher grading and Ki-67 staining and leads to more frequent and earlier local or distant visceral recurrence and shorter survival (Foulkes et al. 2010; Hernandez-Aya et al. 2011). As TNBC tumors lack steroid hormone receptors or HER2 overexpression, systemic treatment is limited to chemotherapy, and – if associated with BRCA mutations – PARP inhibitors (Park et al. 2018).

In order to determine KLK12 mRNA expression levels in TNBC, we developed a sensitive quantitative real-time PCR assay (qPCR) and subsequently analyzed whether KLK12 mRNA expression is associated with established clinical variables such as age, lymph node status, tumor size, and histological grade as well as with disease-free (DFS) and overall survival time (OS) of the patients.

## Methods

### Breast cancer tissues and study population

In the current study, cancerous tissue specimens from 127 patients afflicted with triple-negative breast cancer (TNBC), undergoing mastectomy or breast conservation surgery at the Department of Obstetrics and Gynecology, Klinikum rechts der Isar, Technical University of Munich (TUM), between 1988 and 2012, were included. All tumor specimens were stored in liquid nitrogen immediately after surgery and histologically confirmed by pathologists for the estimation of tumor size, grade, axillary lymph node status, TNM stage, as well as for the lack of expression of estrogen receptor (ER), progesterone receptor (PR) and lack of overexpression of the human epidermal growth factor receptor 2 (HER2). No data were available concerning Ki67 expression.

The patients were 30–96 years of age (median, 55 years) and tumor size ranged from 0.5 to 11 cm with a median of 2.55 cm. Of the 127 TNBC cases, 116 (91%) were categorized as invasive ductal carcinoma, whereas the rest were rare subtypes like medullary carcinoma, lobular carcinoma, and other histologic subtypes. The follow-up time of patients after primary tumor resection ranged from 4 to 286 months for OS (median, 79 months) and from 3 to 269 months for DFS (median, 71 months). In 2 of 127 cases for overall survival (OS) and 4 cases for disease-free survival (DFS), no follow-up information was available.

### Real-time polymerase chain reaction

Purified RNA (and cDNA) was stored at − 80 °C until further use. The concentration and purity of RNA were estimated by the Nano Drop 2000c spectrophotometer and the Nano Drop 2000/2000c software (Thermo Fisher Scientific, Wilmington, USA). With respect to RNA extraction, reverse transcription, first-strand cDNA synthesis and quantitative real-time polymerase chain reaction (qPCR), a comprehensive description has been previously published by Ahmed et al. (2016). KLK12 mRNA expression was assessed by qPCR on an Agilent MX3005P system (Agilent, Darmstadt). Assays have been established in-house using the Universal ProbeLibrary (Roche, Penzberg, Germany). Hypoxanthine guanine phosphoribosyl transferase 1 (HPRT1) was used as the reference gene (for details see Ahmed et al. (2016)).

For quantitation of KLK12 mRNA expression, a commercially available TaqMan gene expression assay (Hs00377603_m1; ThermoFisher, Schwerte, Germany) was used. This assay detects all known KLK12-derived transcripts including those encoding the full length protein (NM_019598.2, variant 1; NM_145894.1, variant 2).

Standard dilution series were employed to determine the reaction efficiency and sensitivity (Bustin and Nolan 2013). Threshold cycles (Ct) were utilized to calculate the grade of KLK12 mRNA expression compared to the respective housekeeping gene HPRT1 by relative quantification using the 2exp-ΔΔCt method (Pfaffl 2012).

Among the 127 cases, 10 cases were excluded due to very low RNA concentrations. Additionally, in view of the detection limitation and variations of tissue qualities, samples had to meet the following quality criteria: a Ct value for HPRT of < 35; a 2exp-∆∆Ct error progression% of < 30% even after repetition, and a % STDEV of the 2 exp-∆∆Ct for 2 valid runs of < 47.1% (Ahmed et al. 2016). Based on this, one further case was excluded from the current study and, thus, finally 116 TNBC cases were included to analyze the clinical impact of KLK12 mRNA levels on patients with TNBC.

### Statistical analysis

The SPSS statistical analysis software (version 20.0; SPSS Inc., Chicago, IL, USA) was employed for statistical analysis. The association of KLK12 mRNA levels with clinical parameters was analyzed using Chi-square tests. The predictive ability of clinical variables and KLK12 mRNA expression was studied by univariate and multivariate proportional hazards regression analyses. In addition, Kaplan-Meier survival analysis was performed to evaluate the prognostic potential of KLK12 mRNA for DFS and OS of TNBC patients. *P*-values < 0.05 were considered statistically significant.

## Results

### KLK12 mRNA expression in tumor tissues of TNBC patients

KLK12 mRNA expression levels were determined by qPCR in a homogenous cohort of 116 patients with triple-negative breast cancer. The values of KLK12 mRNA expression normalized to mRNA expression of HPRT1 range from 0.00 to 0.38 (median 0.00; mean 0.0067). Positive, but low KLK12 mRNA levels were detected in 54 of 116 cases (47%), whereas the rest of the tumor tissues (*n* = 62, 53%) displayed negative KLK12 expression. Based on this, KLK12 was dichotomized into positive expression versus negative expression. Table 1 summarizes the analysis of KLK12 mRNA expression in relation to the established clinicopathological variables, including age, lymph node status, tumor size, and histological grade. No significant association was observed between KLK12 levels and any of these parameters.

**Table 1:** Correlation of KLK12 mRNA expression with clinicopathological variables in patients with triple-negative breast cancer

Clinical parameters	No. of patients	KLK12^a^
		Negative/Positive
Age		*p* = 0.487
≤ 60 years	62	35/27
> 60 years	54	27/27
Lymph node status		*p* = 0.214
N0	63	37/26
N1/N2/N3	53	25/28
Tumor size		*p* = 0.815
≤ 20 mm	31	17/14
> 20 mm	84	44/40
Histological grade		*p* = 0.405
Grade II	20	9/11
Grade III	96	53/43

Due to missing values, numbers do not always add up to *n* = 116

^a^ Categorized into positive- and negative-expressing groups

### KLK12 mRNA expression and established clinicopathological variables in relation to DFS and OS of TNBC patients

By univariate Cox regression analysis (Table 2; observation time: 15 years), age was the only univariate predictor of the established clinical parameters both for DFS and OS of TNBC patients (*p* = 0.001, *p* <  0.001, respectively). Positive KLK12 mRNA expression was shown to be significantly associated with shortened DFS (hazard ratio [HR] = 2.12, 95% CI = 1.19–3.78, *p* = 0.010) as well as OS (HR = 1.91, 95% CI = 1.04–3.50, *p* = 0.037). In both cases, the HR is around 2, indicating an about two-fold increased probability of disease progression and higher risk of cancer-related death in the KLK12 expressing group.

**Table 2:** Univariate Cox regression analysis of KLK12 mRNA expression in relation to patients’ outcome in triple-negative breast cancer

Clinical parameters	OS			DFS		
	No.^a^	HR (95% CI)^b^	*p*	No.^a^	HR (95% CI)^b^	*p*
Age			**< 0.001**			**0.001**
≤ 60 years	67	1		67	1	
> 60 years	58	2.96 (1.63–5.36)		56	2.45 (1.42–4.23)	
Lymph node status			*0.055*			0.118
N0	70	1		69	1	
N1/N2/N3	55	1.74 (0.99–3.07)		54	1.53 (0.90–2.59)	
Tumor size			0.117			0.204
≤ 20 mm	33	1		33	1	
> 20 mm	91	1.83 (0.86–3.92)		89	1.54 (0.79–2.98)	
Histological grade			0.915			0.600
II	21	1		21	1	
III	104	1.04 (0.49–2.23)		102	1.22 (0.58–2.59)	
KLK12 mRNA^c^			**0.037**			**0.010**
Negative	61	1		59	1	
Positive	54	1.91 (1.04–3.50)		54	2.12 (1.19–3.78)	
KLK12 mRNA pos^d^			**0.047**			*0.051*
Low	16	1		16	1	
High	38	2.98 (1.02–8.74)		38	2.64 (1.00–6.96)	

Significant *p*-values (*p* ≤ 0.05) are indicated in bold, trends towards significance (*p* ≤ 0.06) in italics

Due to missing values, numbers do not always add up to *n* = 125 (OS) and *n* = 123 (DFS), when the whole cohort is analyzed

^a^ Number of patients

^b^*HR* Hazard ratio (*CI* Confidence interval) of univariate Cox regression analysis

^c^ Dichotomized into positive and negative expression

^d^ Positive KLK12 mRNA values were dichotomized into low and high expression by the 30th percentile

**Fig. 1:**
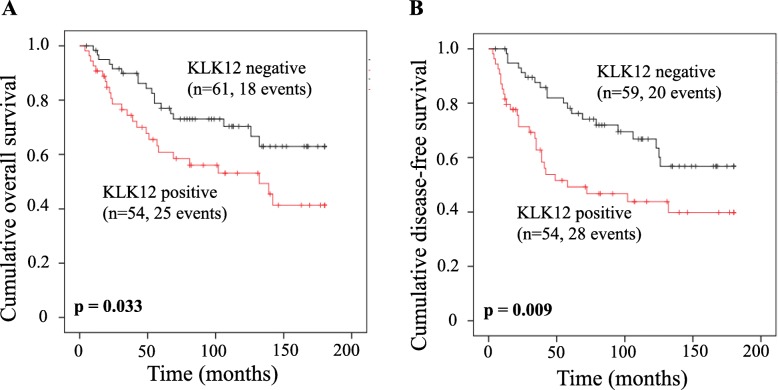
Overall survival (OS) and disease-free survival (DFS) of patients with triple-negative breast cancer according to KLK12 mRNA expression in primary tumor tissues. Patients with positive KLK12 mRNA expression had a significantly shortened OS (**a**) and DFS (**b**) than those with negative KLK12 mRNA expression (*p* = 0.033 and *p* = 0.009, respectively). Survival curves were drawn according to the Kaplan-Meier survival analysis. *P* values were calculated via the log-rank algorithm

The impact of KLK12 mRNA expression on clinical outcome was further validated by the Kaplan-Meier survival analysis (Fig. 1), demonstrating that positive KLK12 mRNA expression is significantly correlated with poor prognosis of TNBC patients.

**Fig. 2:**
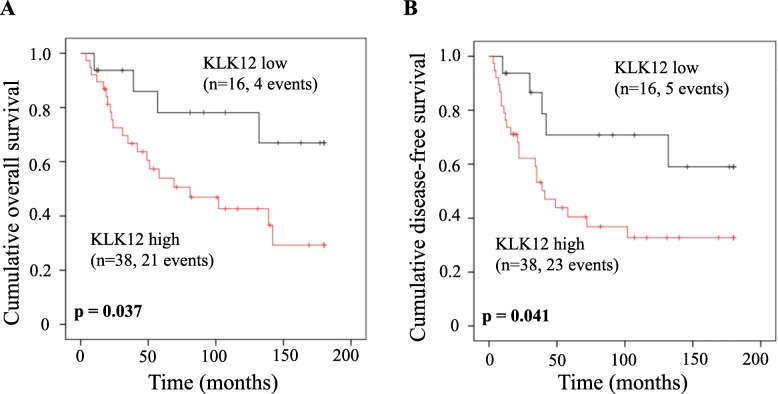
Overall survival (OS) and disease-free survival (DFS) of triple-negative breast cancer patients in the sub-group of patients displaying positive KLK12 mRNA expression in primary tumor tissues. Patients with elevated KLK12 mRNA expression levels display a significantly worse OS (**a** Kaplan-Meier analysis, *p* = 0.037) and DFS (**b***p* = 0.041), respectively, compared to patients with low KLK12 mRNA expression levels

The impact of KLK12 mRNA expression was also evaluated in the subgroup of patients displaying positive expression of KLK12 mRNA (*n* = 54). After dichotomization in high versus low expression, we observed that elevated KLK12 mRNA levels compared to lower KLK mRNA levels were associated with worse prognosis (OS: HR = 2.98, 95% CI = 1.02–8.74, *p* = 0.047; DFS: HR = 2.64, 95% CI = 1.00–6.96, *p* = 0.051; Table 2). These findings are visualized by the respective Kaplan-Meier survival curves in Fig. 2 and indicate that there may be an association between the amount of KLK12 mRNA expression and length of OS and DFS, respectively.

The independent relationship of KLK12 mRNA levels with patient outcome was assessed in the complete cohort using multivariable Cox regression analysis, including the clinical variables age, lymph node status, tumor size, and histological grade (base model). In the base model, age remains a prognostic indicator for both DFS (*p* <  0.001) and OS (*p* <  0.001). KLK12 mRNA expression significantly contributes to the base model and represents an independent unfavorable predictive marker for DFS (HR = 2.33, 95% CI = 1.28–4.24, *p* = 0.006), while only showing a trend towards significance in case of OS (HR = 1.80, 95% CI = 0.96–3.38, *p* = 0.066) (Table 3).

**Table 3:** Multivariable Cox regression analysis of KLK12 mRNA expression in relation to patients’ outcome in triple-negative breast cancer

Clinical parameters	OS			DFS		
	No.^a^	HR (95% CI)^b^	*p*	No.^a^	HR (95% CI)^b^	*p*
Age			**< 0.001**			**< 0.001**
≤ 60 years	60	1		60	1	
> 60 years	54	3.77 (1.93–7.37)		52	3.16 (1.72–5.80)	
Lymph node status			**0.036**			0.065
N0	62	1		61	1	
N1/N2/N3	52	1.96 (1.05–3.68)		51	1.73 (0.97–3.11)	
Tumor size			0.241			0.402
≤ 20 mm	31	1		31	1	
> 20 mm	83	1.64 (0.72–3.73)		81	1.35 (0.67–2.74)	
Histological grade			0.465			0.255
II	20	1		20	1	
III	94	1.36 (0.60–3.10)		92	1.60 (0.71–3.62)	
KLK12 mRNA^c^			0.066			**0.006**
Negative	60	1		58	1	
Positive	54	1.80 (0.96–3.38)		54	2.33 (1.28–4.24)	

The biological marker KLK12 mRNA was added to the base model of clinical parameters: age, residual tumor mass, and ascites fluid volume. Significant *p*-values (*p* ≤ 0.05) are indicated in bold

^a^ Number of patients

^b^*HR* Hazard ratio (*CI* Confidence interval) of multivariable Cox regression analysis;

^c^ Dichotomized into positive and negative expression

## Discussion

In the present study, we assessed KLK12 mRNA expression levels by qPCR in 116 TNBC tumors, and analyzed their association with established clinical variables as well as survival.

Using qPCR, we failed to detect mRNA expression of the KLK12 gene in nearly half of tissue samples (62/116, 53%), while the expression levels remained low in the other TNBC samples. A lack of KLK12 mRNA expression in a large proportion of the breast tumors examined (12/17) had already been noted by Yousef and collaborators (2000). Similarly, no KLK12 mRNA expression was observed in tumor tissue samples from 32 patients with advanced high-grade serous ovarian cancer (own unpublished data). In contrast, moderate to strong expression was seen in tumor tissues from patients with gastric cancer (Zhao et al. 2012). Moreover, the stomach is one of the few healthy tissues that strongly express the KLK12 gene which is not the case in the breast and the ovary (Shaw and Diamandis 2007). Thus, it is possible that tissue-specific factors influence and condition KLK12 gene expression in both healthy and tumor tissues. However, KLKs are known to be dysregulated in many tumors in comparison with the healthy tissues (Mavridis and Scorilas 2010; Tailor et al. 2018). KLK12 is one of the 9 KLK genes (namely, KLK1, KLK2, KLK6, KLK7, KLK9, KLK10, KLK11, KLK12 and KLK14) whose mRNA levels were found to be significantly down-regulated in malignant breast tissues compared to normal breast tissues (Mange et al. 2008). A similar observation was made for KLK12 in a recent study comparing breast tumor samples and matched non-tumor samples (Papachristopoulou et al. 2018). How KLK genes are downregulated is not fully elucidated, especially with regard to the KLK12 gene. A number of studies have investigated the relationship between KLK gene methylation and expression (Pampalakis et al. 2006; Pasic et al. 2012). KLK12 was found reactivated in the PC3 prostate cancer cell line following treatment with 5-aza-2′-deoxycytidine (5-aza-dC). However, this gene does not contain CpG islands (Pampalakis et al. 2006), which is in line with the observation that its expression remained unaffected in breast and ovarian cancer cell lines upon 5-aza-dC treatment. This suggests that expression of KLK12 in immortalized cell lines is actually not regulated by methylation, upregulation of KLK12 in PC3 cells may probably result from a side effect of 5-aza-dC. Some studies have also revealed a role of histone modifications in the regulation of KLK gene expression. In breast cancer, constitutive and inducible expression of KLK6 positively correlated with histone H4 acetylation located in KLK6 upstream sequences (Pampalakis and Sotiropoulou 2006; Pampalakis et al. 2009). To our knowledge, no data exists for other KLK genes in breast cancer. Accumulating evidence indicates that microRNAs (miRNAs) are involved in post-transcriptional regulation of several KLK genes in cancer, e.g. KLK6 and KLK10 (Di Meo et al. 2018). However, KLK12 does not have strongly predicted miRNA/KLK interactions (Chow et al. 2008) and no current data support regulation of this gene via direct actions of miRNAs. So, further studies are needed to determine the modalities of KLK12 silencing in TNBC.

The KLK12 gene produces at least five alternative transcripts resulting from 5′ exon extension, exon skipping, or intron retention (Adamopoulos et al. 2018; Kurlender et al. 2005). The classical KLK12 serine protease (248 amino acids [aa]) is encoded by the splicing variant sv2, whereas KLK12sv1 encodes a longer KLK12 isoform (254 aa; the C-terminal 13 aa of sv2 are exchanged by 19 alternative aa), also displaying the catalytic triad. The three other transcripts (sv3, sv4 and sv5) are predicted to encode significantly truncated proteins lacking the potential to display proteolytic activity (Kontos and Scorilas 2017). Our qPCR assay quantifies all KLK12 transcripts thus measuring overall expression of this gene in TNBC. Positive KLK12 mRNA expression was significantly associated with shortened DFS as well as OS in the univariate cox regression analysis and represented an independent unfavorable predictive marker for DFS in TNBC. Such associations have already been observed in other malignancies. KLK12 expression in gastric cancer was found to be significantly and positively associated with higher tumor-node-metastasis (TNM) stage and patients with high KLK12 mRNA expression displayed a significantly poorer 5-year survival rate than those with low KLK12 expression (Zhao et al. 2012). High KLK12 mRNA expression was also described as being an unfavorable prognostic indicator in pulmonary carcinoid (Swarts et al. 2013). These observations are consistent with the currently known functions of the KLK12 protease; indeed, the functional studies performed to date argue for a pro-tumorigenic role of this protease. Blocking KLK12 expression in gastric cancer cells significantly inhibited proliferation by arresting cells in G0/G1 phase (Li and He 2016; Zhao et al. 2012). KLK12 also regulates cell adhesion and migration of endothelial and malignant cells likely through the cleavage of structural components of the extracellular matrix (ECM) (Li and He 2016; Zhao et al. 2012; Kryza et al. 2018). Furthermore, KLK12 has been reported for its proangiogenic effect, thereby displaying a crucial role in the process of cancer. KLK12 may indirectly modulate the bioavailability and/or activity of various growth factors like VEGF165, BMP2 (bone morphogenetic proteins 2), TGF-β1, and FGF-2 (fibroblast growth factor 2), through hydrolyzing proteins of the CCN family (Guillon-Munos et al. 2011), which are matricellular proteins involved in angiogenesis and tumorigenesis (Dallas et al. 2005). Also, KLK12 may modulate the availability of platelet-derived growth factor B (PDGF-B) via cleaving its C-terminal retention motif (Kryza et al. 2014). PDGF-B is an extracellular matrix- or membrane-bound precursor and acts as an autocrine and paracrine growth factor that stimulates tumor growth and angiogenesis.

Two studies have examined expression of distinct KLK12 transcripts in breast cancer (Papachristopoulou et al. 2018; Talieri et al. 2012). KLK12sv3 expression was found more frequently expressed in tumors of lower grade and positive estrogen and progesterone receptor status. Patients with high KLK12sv3 expression levels presented either only longer disease-free survival (Talieri et al. 2012) or both longer disease-free survival and overall survival (Papachristopoulou et al. 2018). So, KLK12sv3 could be regarded as a marker of good prognosis in breast cancer but probably not for TNBC since this transcript is poorly or not expressed in ER and PR negative breast tumors (Papachristopoulou et al. 2018). Under these conditions, it seems likely that the overall KLK12 expression determined in the present study mainly corresponds to the KLK12sv1/2 transcripts encoding KLK12 versions encompassing its complete catalytic triad. Divergences between the prognostic values of transcript coding for a KLK protease and of alternative transcript encoding a truncated form of the protein has already been observed in the lung for the KLK8 gene (Planque et al. 2008). This suggested that the same KLK gene could produce two products with opposite effects on tumor growth and dissemination. Papachristopoulou and collaborators revealed that KLK12sv3 was significantly higher expressed in benign breast tumors than in breast cancers and that KLK12sv3 levels declined in more aggressive forms of breast carcinoma. Taken together, all data suggest that the protease KLK12 would play a pro-tumorigenic role in breast cancer whereas KLK12vs3 (encoding a truncated protein lacking a functional catalytic triad) would have a tumor suppressor role. Functional studies would be necessary to examine this hypothesis.

KLK12 is a trypsin-like serine protease secreted as an inactive pro-enzyme which is able to autoactivate to acquire enzymatic activity. KLK12 is likely involved in enzymatic cascades as this protease is able to activate KLK11 zymogen in vitro (Yoon et al. 2007). Interestingly, we found a positive moderately high correlation between the mRNA expression of KLK12 and that of KLK10 and KLK11 in our cohort (data not shown). Further investigations are required to determine whether KLK12 may represent a physiological activator of KLK10/KLK11 in some TNBC tissues.

## Conclusion

In conclusion, our results revealed that positive KLK12 expression is remarkably associated with shortened DFS and OS, suggesting that KLK12 mRNA might be a prognostic biomarker and therapy target in triple-negative breast cancer. The comparison of our data with those of literature suggests ambivalent roles of KLK12 products, some being pro- and others anti-tumorigenic.

## Data Availability

This article is original and has not been published elsewhere. Patient-related data are available via the Ethics Committee of the Medical Faculty of the Technical University of Munich, Ismaninger Str. 22, 81675 Munich, Germany, for researchers who meet the criteria for access to confidential data. According to the Bavarian Data Protection Authority (BayLDA) and the General Data Protection Regulation (GDPR), patient-related data will only be made available to third parties after double-pseudonymization, undertaken by the Dept. of Medical Statistics and Epidemiology, Technical University of Munich.

## Abbreviations

5-aza-dC: 5-aza-2′-deoxycytidine
aa: Amino acids
BMP2: Bone morphogenetic proteins 2
Ct: Threshold cycles
DFS: Disease-free survival
ECM: The extracellular matrix
ER: Estrogen receptor
FGF-2: Fibroblast growth factor 2
HER2: Human epidermal growth factor receptor 2
HPRT1: Hypoxanthine guanine phosphoribosyl transferase 1
HR: Hazard ratio
KLK: Kallikrein-related peptidase
KLK1: Tissue kallikrein
MVEC: Microvascular endothelial cells
OS: Overall survival
PDGF-B: Platelet-derived growth factor B
PR: Progesterone receptor
qPCR: Quantitative real-time PCR
TNBC: Triple-negative breast cancer
TNM: Tumor-node-metastasis
